# Supplementary figures and images for: LF4/MOK and a CDK-related kinase regulate the number and length of cilia in Tetrahymena
Source: PLoS Genet. 2019 Jul 24;15(7):e1008099. doi: 10.1371/journal.pgen.1008099 (PMC6682161; doi:10.1371/journal.pgen.1008099)

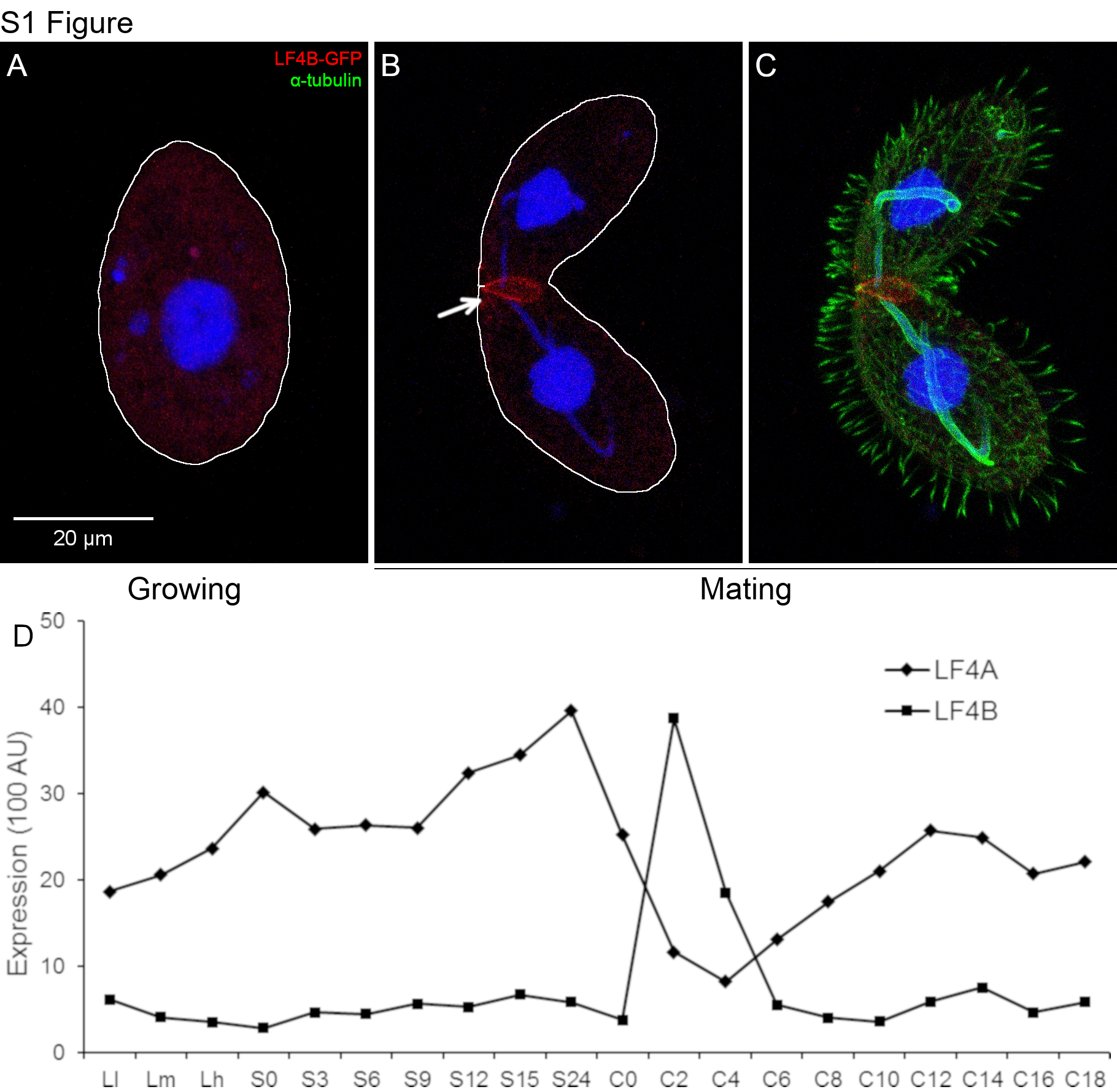

Supplement: S1 Fig — (A-C) Cells expressing LF4B-GFP (the tag added by engineering the native locus) analyzed by immunofluorescence using anti-GFP antibodies (red) 12G10 anti-α-tubulin (green only in panel C) and DAPI (Blue). (A) A vegetatively growing cell. Note an absence of a GFP signal above the typical background. (B-C) A conjugating pair. Note that LF4B-GFP localizes to the junction between the two mating cells. (D) Expression profiles of mRNAs for LF4A (TTHERM_00058800) and LF4B (TTHERM_00822360) obtained from the Tetrahymena Functional Genomics database (http://tfgd.ihb.ac.cn/search/detail/gene/TTHERM_00822360). The levels of mRNA at the following conditions are shown: L-l, L-m and L-h: vegetatively growing cells collected at ~1x10^5^ cells/ml, ~3.5x10^5^cells/ml and ~1x10^6^ cells/ml. S-0, S-3, S-6, S-9, S-12, S-15 and S-24: cells starved for 0, 3, 6, 9, 12, 15 and 24 hours. C-0, C-2, C-4, C-6, C-8, C-10, C-12, C-14, C-16 and C-18: conjugating cells collected at 0, 2, 4, 6, 8, 10, 12, 14, 16 and 18 hours after initiation of conjugation by mixing different mating types. (TIF) [file pgen.1008099.s001.tif]

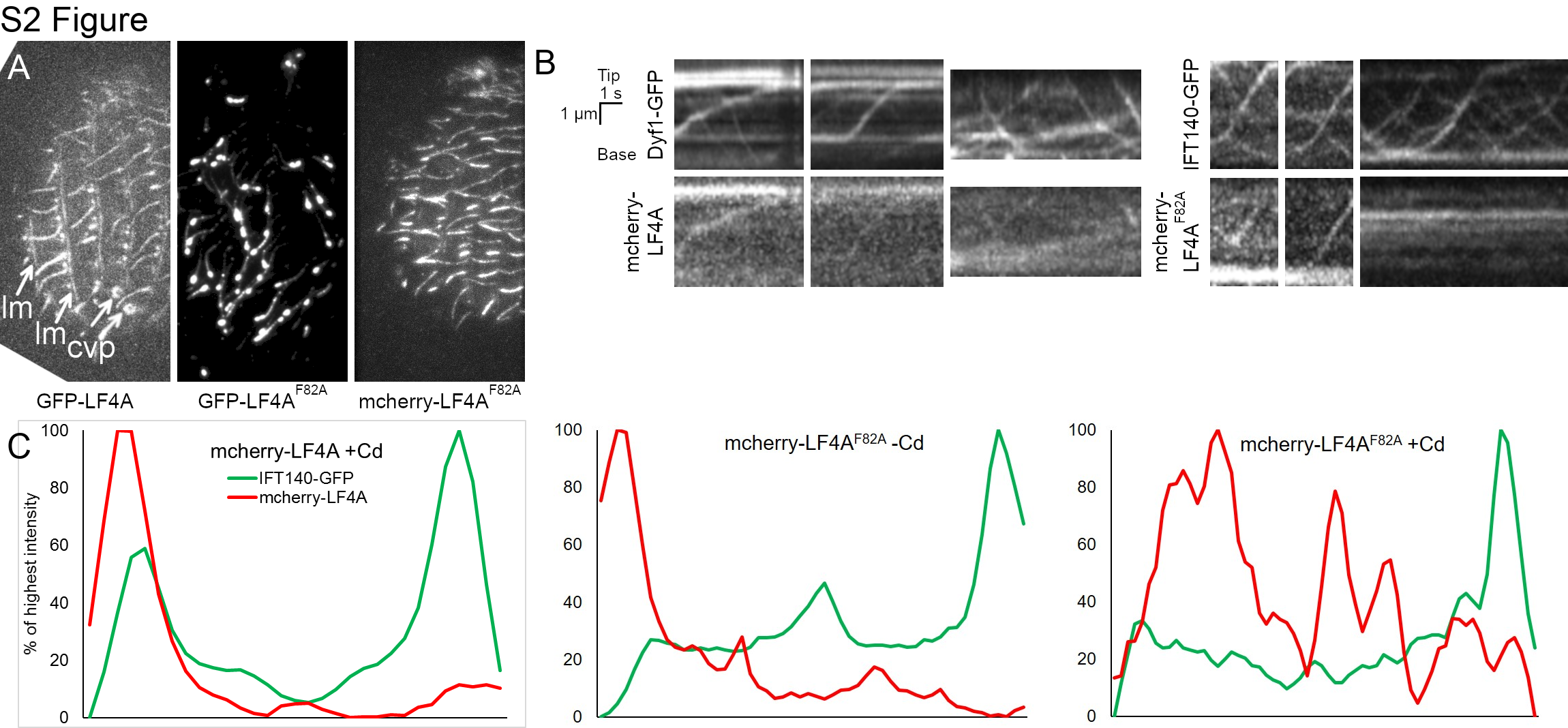

Supplement: S2 Fig — (A) TIRF images of live cells overexpressing GFP-LF4A (left), kinase-weak GFP-LF4AF82A variant (middle), and kinase weak mCherry-LF4AF82A. Overexpressed GFP-LF4A (right panel) localized to the bases of cilia and along cilia but also near the microtubule-rich structures in the cell body including longitudinal microtubules (lm), and contractile vacuole pores (cvp). The kinase-weak GFP-LF4AF82A is enriched at the tips of cilia while mCherry-LF4AF82A is distributed uniformly along cilia. (B) Kymographs that document co-migration of IFT proteins (GFP-DYF1 or IFT140-GFP, top) and either mCherry-LF4A or mCherry-LF4AF82A after induction with Cd2+ (3 hours). (C) Signal intensity profiles of single cilia in cells expressing either mCherry-LF4 or mCherry-LF4F82A (red) and IFT140-GFP (green). The base is on the left and the tip is on the right side of each profile. Note that the active kinase is enriched at the base (left profile). The weak kinase is enriched at the base and spread along the cilium length when overproduced but does not accumulate at the tip. The pattern distribution of IFT140 is similar in all backgrounds and conditions, with enrichment at the tip. (TIF) [file pgen.1008099.s002.tif]

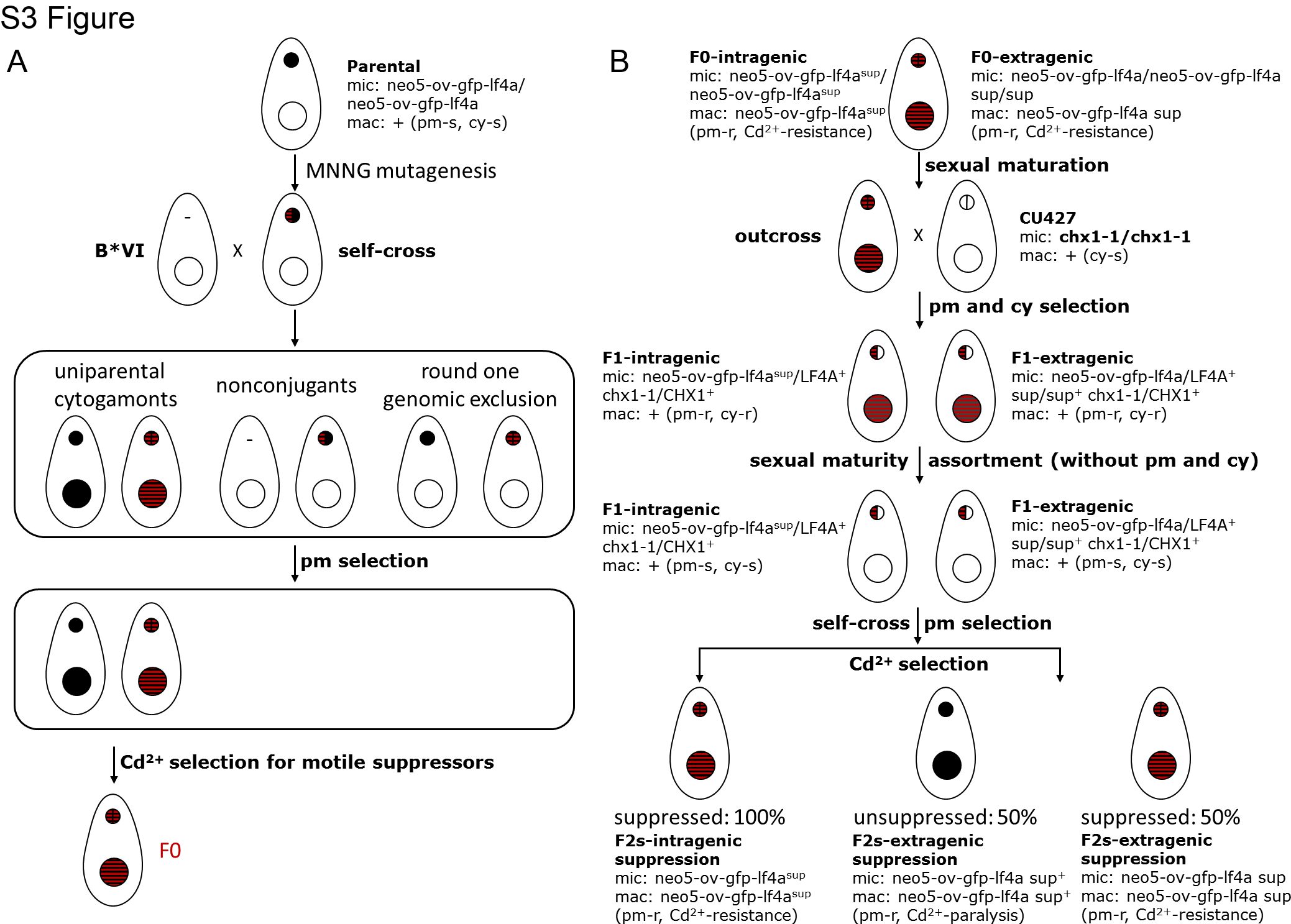

Supplement: S3 Fig — (A) Steps involved in generation and isolation of the suppressor F0s. A heterokaryon with the ovGFP-LF4A transgene in the micronucleus (solid black) was subjected to mutagenesis with nitrosoguanidine. The mutagenized heterokaryon was subjected to a self-cross (uniparental cytogamy) that involves mating to a star strain that lacks a functional micronucleus. The outcome includes the desired self-cross progeny (uniparental cytogamonts, typically a few % of the conjugated pairs), cells that failed to undergo conjugation (nonconjugants) and round one genomic exclusion, the most common outcome of such a cross (typically >95%). The uniparental cytogamy progeny were selected with paromomycin (pm) as they expressed the transgene in the macronucleus. The suppressor F0s were then isolated by collecting cells that remained mobile after overnight Cd2+ exposure. (B) Steps used to determine whether the suppression is intragenic or extragenic. Each suppressor F0 clone underwent sexual maturation and was mated to CU427, a strain with a micronucleus carrying a homozygous chx1-1 allele (resistance to cycloheximide cy) and a wild-type macronucleus. The outcross progeny was selected with cy and pm. F1 clones underwent phenotypic assortment to pm-s and become sexually mature. The pm-s F1 clone was then subjected to self-cross (short-circuit genomic exclusion) and the pm-r F2 clones were obtained. A number of F2 clones of each suppressor were tested for suppression by Cd2+ treatment. An intragenic suppressor gives only suppressed F2 clones. An extragenic suppressor gives both unsuppressed (paralyzed) and suppressed F2 progeny. (TIF) [file pgen.1008099.s003.tif]

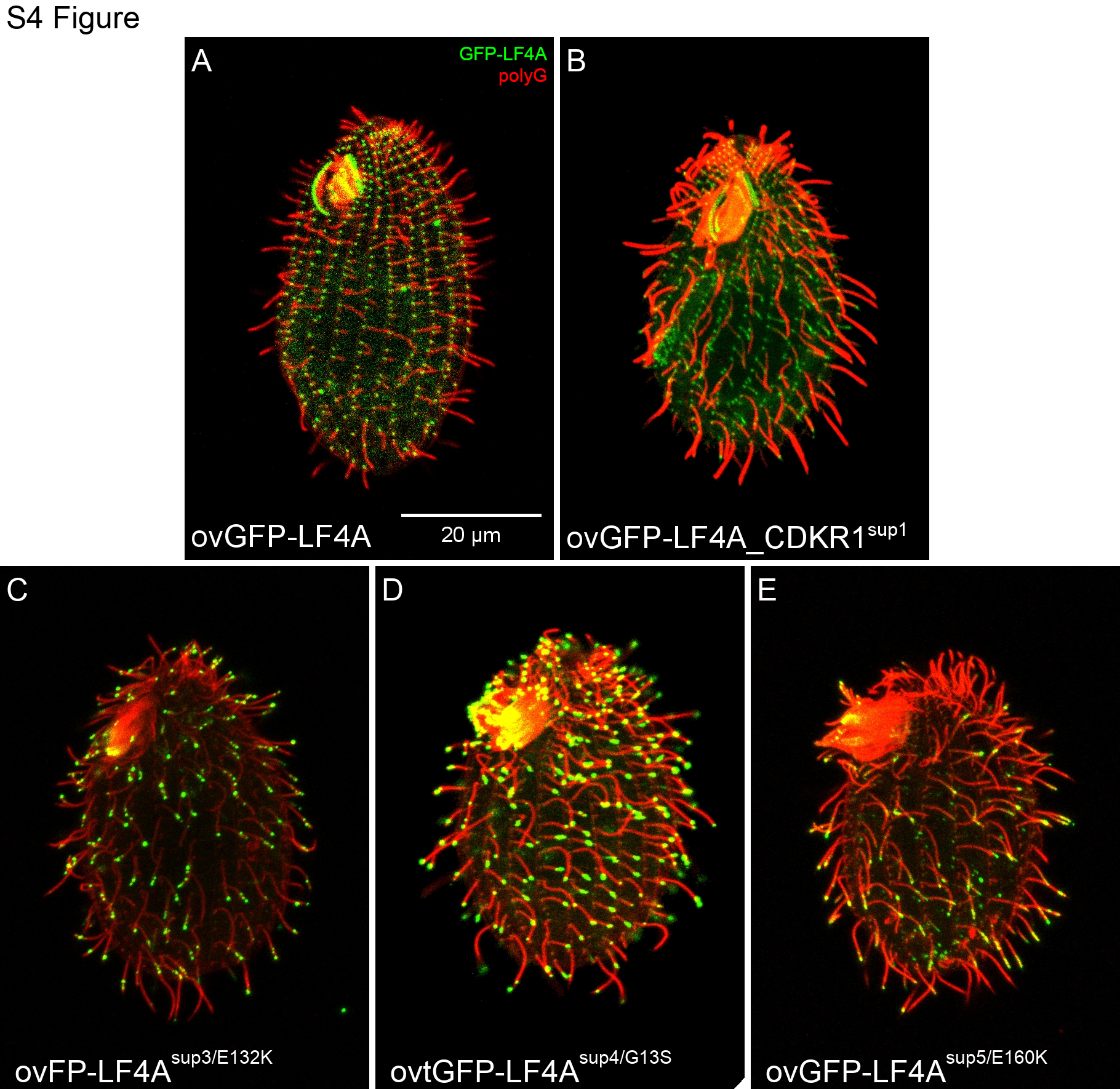

Supplement: S4 Fig — Self-cross progeny at a control background of GFP-LF4A overexpression (A), the extragenic suppressor SUP1 (B) and three intragenic suppressors SUP3, SUP4 and SUP5 (C-E). All cells were subjected to a 6-hours Cd2+ exposure prior to immunofluorescence assay and showed the GFP signal (green) and were stained with anti-polyG antibodies (red). (TIF) [file pgen.1008099.s004.tif]

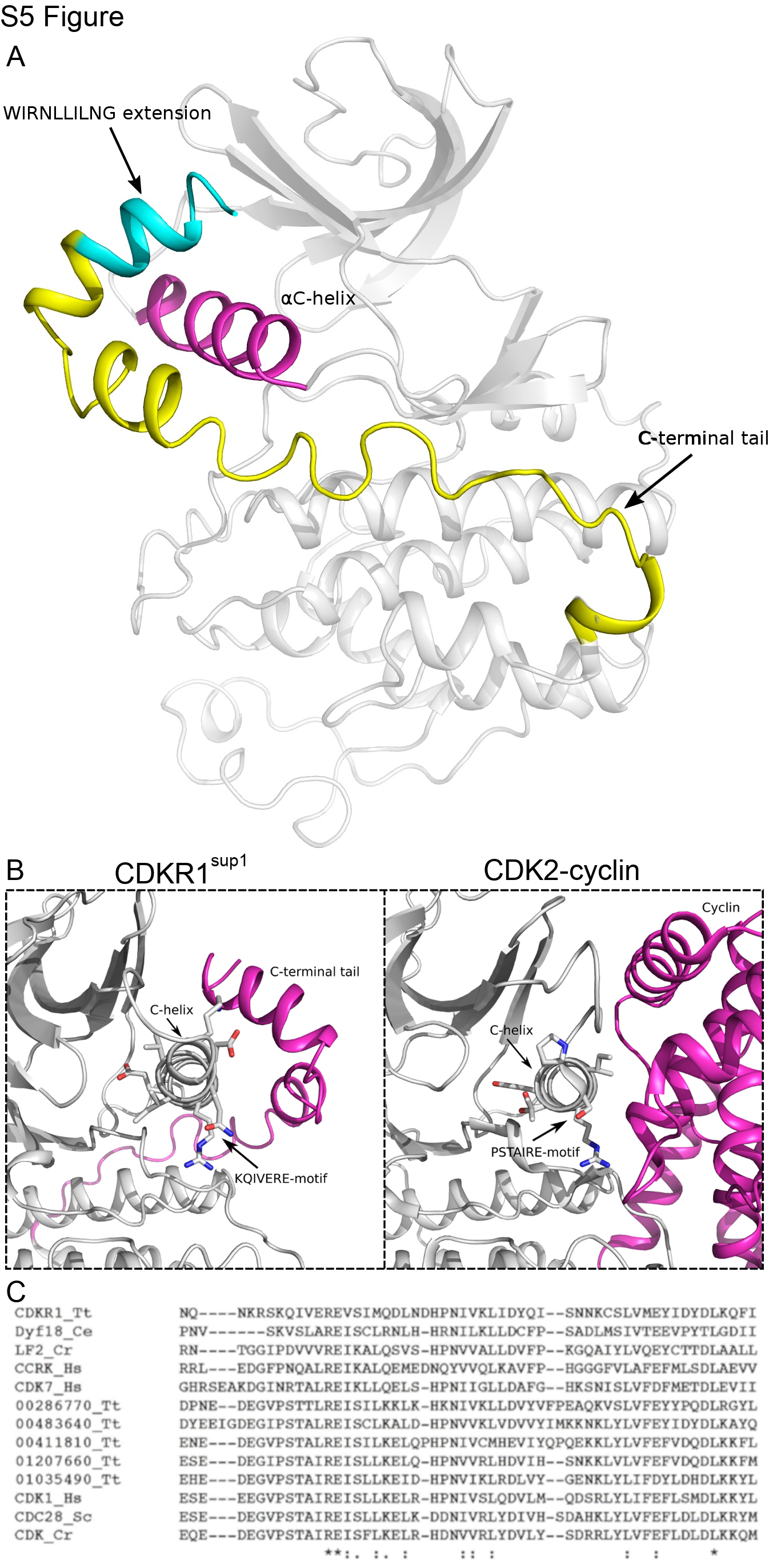

Supplement: S5 Fig — (A) Predicted structure of CDKR1sup1 with a C-terminal tail (with a WIRNLLILNG extension) forming two helical segments on the top of C-helix. (B) 3D view comparison of the C-helix (and cyclin-CDK interface) of CDKR1sup1 and a CDK2. A PSTAIRE sequence in the canonical CDKs lies at the interface of the cyclin-CDK complex and corresponds to the C-helix in the CDKs. The equivalent positions in CDKR1 are KQIVERE. (C) A sequence alignment of fragments of CDK and CDK-related kinases. (TIF) [file pgen.1008099.s005.tif]

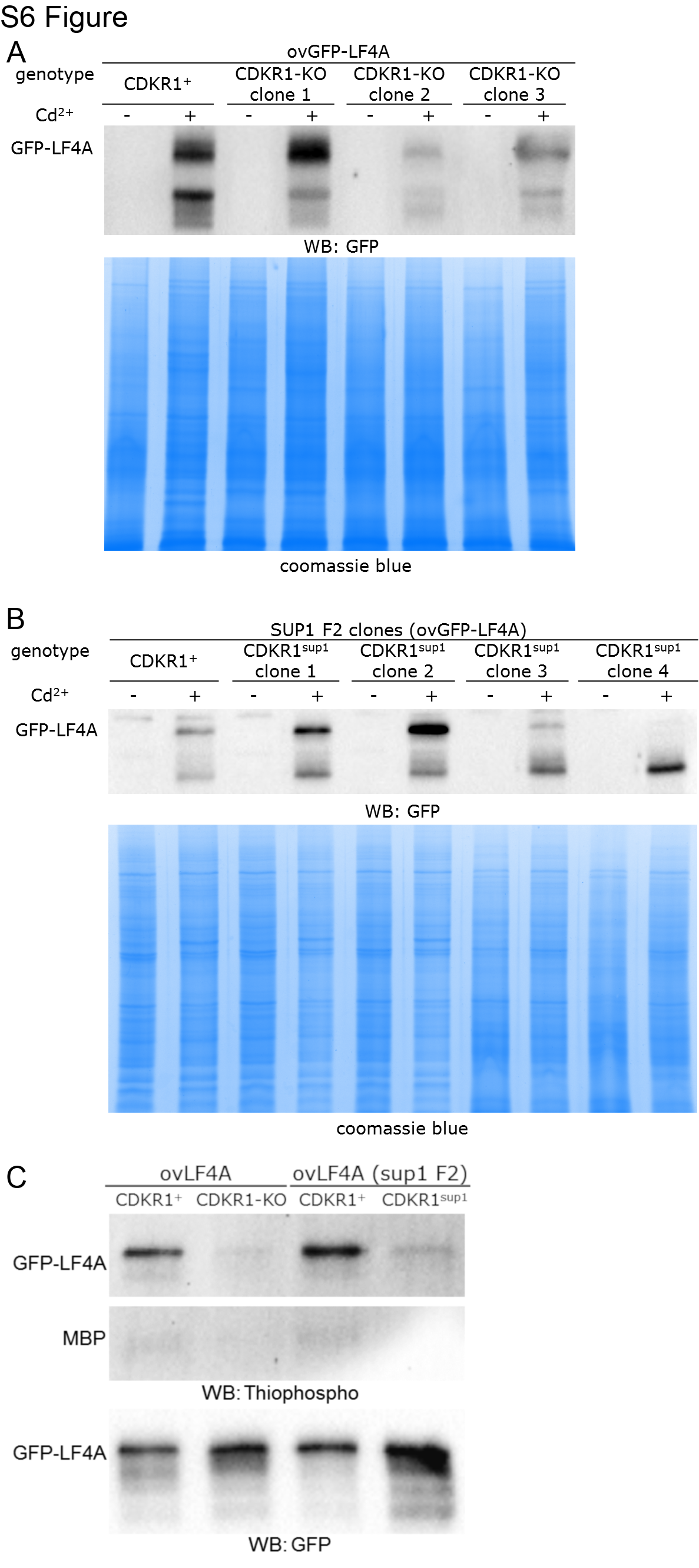

Supplement: S6 Fig — (A) A comparison of the levels of GFP-LF4A in whole cell lysates of several F2 clones derived from the same F1, with or without a 6-hour Cd2+ exposure. An ovGFP-LF4A_CDKR1+ control strain and three ovGFP-LF4A_CDKR1-KO F2s were analyzed. (B) A comparison of the levels of GFP-LF4A in whole cell lysates of the F2 progeny clones of the extragenic suppressor SUP1, with or without a 6-hour Cd2+ exposure. An unsuppressed (ovGFP-LF4A_CDKR1+) strain and four suppressed (ovGFP-LF4A_CDKR1sup1) strains were analyzed. Faster-migrating bands represent degradation products of GFP-LF4A. (C) In vitro kinase assays show that loss-of-function of CDKR1 results in a reduced kinase activity of overproduced GFP-LF4A against itself and MBP. The top panel is a western blot that reveals the signal of thiophosphorylated substrates. The bottom panel is a western blot that documents the levels of GFP-LF4A in the reactions using anti-GFP antibodies. (TIF) [file pgen.1008099.s006.tif]
